# Induction of Apoptosis and Cell Cycle Blockade by Helichrysetin in A549 Human Lung Adenocarcinoma Cells

**DOI:** 10.1155/2013/857257

**Published:** 2013-03-03

**Authors:** Yen Fong Ho, Saiful Anuar Karsani, Wai Kuan Yong, Sri Nurestri Abd Malek

**Affiliations:** Institute of Biological Sciences, Faculty of Science, University of Malaya, 50603 Kuala Lumpur, Malaysia

## Abstract

Researchers are looking into the potential development of natural compounds for anticancer therapy. Previous studies have postulated the cytotoxic effect of helichrysetin towards different cancer cell lines. In this study, we investigated the cytotoxic effect of helichrysetin, a naturally occurring chalcone on four selected cancer cell lines, A549, MCF-7, Ca Ski, and HT-29, and further elucidated its biochemical and molecular mechanisms in human lung adenocarcinoma, A549. Helichrysetin showed the highest cytotoxic activity against Ca Ski followed by A549. Changes in the nuclear morphology of A549 cells such as chromatin condensation and nuclear fragmentation were observed in cells treated with helichrysetin. Further evidence of apoptosis includes the externalization of phosphatidylserine and the collapse of mitochondrial membrane potential which are both early signs of apoptosis. These signs of apoptosis are related to cell cycle blockade at the S checkpoint which suggests that the alteration of the cell cycle contributes to the induction of apoptosis in A549. These results suggest that helichrysetin has great potentials for development as an anticancer agent.

## 1. Introduction

**Figure 1:**
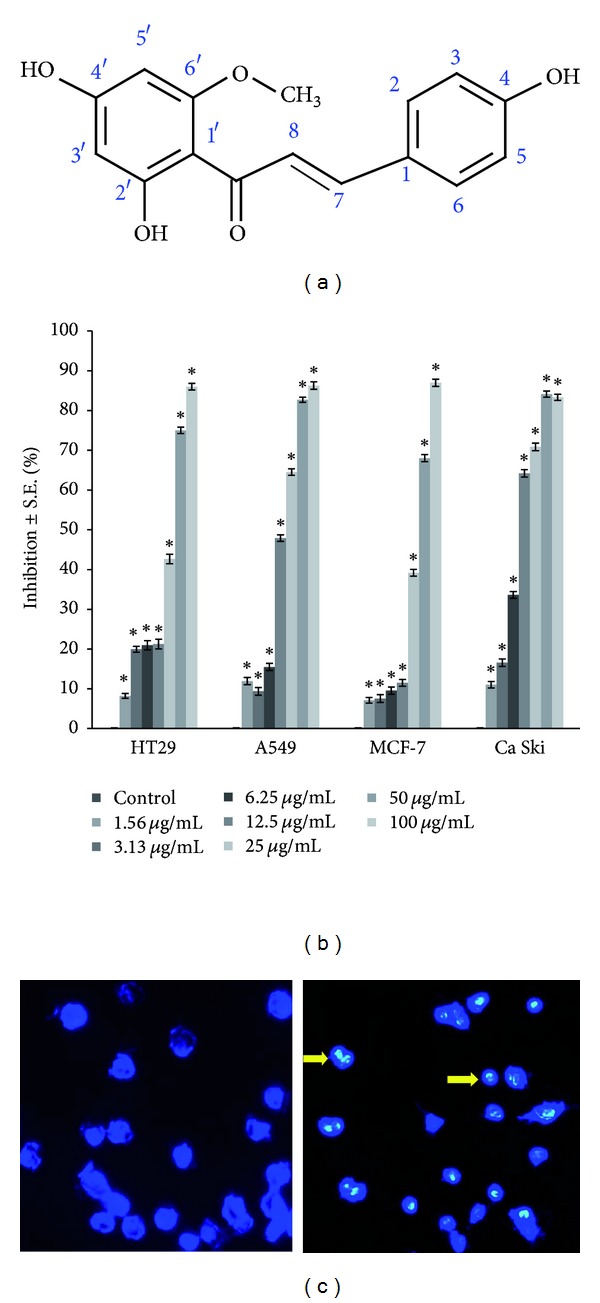
(a) Structure of helichrysetin. (b) HT-29, A549, MCF-7, and Ca Ski cells were treated with different concentrations of helichrysetin for up to 72 hours, and the percentage of inhibition was evaluated using MTT assay. The results are shown as mean ± S.E. and *P* < 0.05 was regarded as statistically significant compared to the untreated control. The percentage of inhibition in untreated control was normalized to 0%. (c) DAPI staining on untreated A549 cells (left) and helichrysetin-treated A549 cells (right) at 40x magnification. Nuclear fragmentation is indicated by bright blue cells (arrow).

Cancer is a disease caused by the uncontrolled growth of abnormal cells in the body. Lung cancer is one of the most commonly diagnosed cancers worldwide making up 12.7% of all cancer cases. It is also the most common cause of cancer death accounting for 18.2% of all cancer associated deaths [1]. Available literature suggested that natural compounds can be effective in cancer therapy [2, 3]. Helichrysetin, 2′,4,4′-trihydroxy-6′-methoxy chalcone (Figure 1(a)), is a naturally occurring chalcone that is found in the flower of *Helichrysum odoratissimum* [4] and the seeds of the *Alpinia sp. *such as *Alpinia blepharocalyx* [5], *Alpinia katsumadai* [6], and *Alpinia galanga *[7]. Chalcones substituted with OH groups exhibit maximum *in vitro* cytotoxicity against tumour cells and increase in antitumour activity [8]. 

Previous studies have reported that helichrysetin possessed antiproliferative and cytotoxic activity towards murine carcinoma and human fibrosarcoma [5], human cervical adenocarcinoma [9], human liver cancer and human breast cancer [6], and human colon sarcoma cell lines [10]. In addition, helichrysetin has also been shown to possess antiplatelet [11] and antioxidant activities [9]. These studies demonstrated the potential use of helichrysetin as an anticancer agent. However, the mechanism of cell death triggered by helichrysetin has not yet been elucidated.

One of the hallmarks of cancer is the resistance of cancer cells towards apoptosis which contributes to the ineffectiveness of anticancer therapies [12]. Apoptosis is characterized by several biochemical and morphological events, such as nuclear fragmentation, internucleosomal DNA fragmentation [13], cell shrinkage [14], chromatin condensation [15], formation of apoptotic bodies, loss of plasma membrane asymmetry [16], and disruption of mitochondrial membrane [17]. In an attempt to understand the mechanism(s) of action involved, we have investigated the effect(s) of helichrysetin on the viability of selected cancer cell lines. Furthermore, we elucidated, for the first time, the biochemical and molecular mechanisms of apoptosis in cancer cells caused by helichrysetin. Our results showed that helichrysetin inhibits the growth of the selected cancer cells through the induction of apoptosis and cell cycle blockade. 

## 2. Materials and Methods 

### 2.1. Helichrysetin and Standard Drug

Helichrysetin was obtained from BioBioPha Co., Ltd. (Yunnan, China). Helichrysetin was dissolved in dimethyl sulfoxide (DMSO) for all the treatments in this study. Doxorubicin (Sigma) was used as positive control in this study.

### 2.2. Cell Culture

Human cervical carcinoma (Ca Ski), human lung adenocarcinoma (A549), and human breast adenocarcinoma (MCF-7) cells were acquired from American Type Culture Collection (ATCC, USA). The cells were cultured in RPMI-1640 (Roswell Park Memorial Institute), 10% fetal bovine serum (FBS), 2% penicillin/streptomycin, and 1% of amphotericin-B. Human colon adenocarcinoma (HT-29) cells were grown in McCoy's 5A medium, 10% fetal bovine serum (FBS), 2% penicillin/streptomycin, and 1% of amphotericin-B. Cells were maintained in humidified 5% CO_2_ atmosphere at 37°C.

### 2.3. MTT Assay

Cells at a density of 3 × 10^4^ cells/mL were plated onto sterile culture plates. The plates were incubated for 24 hours to allow adherence of cells. The media was removed, and 150 *μ*L of fresh media containing different concentrations of helichrysetin was added. Doxorubicin was added to the plate as positive control. The plates were incubated for 24, 48, and 72 hours at 37°C and 5% CO_2_. MTT assay was performed as described by Mosmann [18] with modifications. Next, 20 *μ*L of MTT solution (Sigma) was added to each well and incubated for 4 hours at 37°C and 5% CO_2_. The media containing MTT was discarded. 150 *μ*L of DMSO was added to dissolve the formazan crystals in every well. Absorbance was then measured at 570 nm and 630 nm as background using a microplate reader (Synergy H1 Hybrid). The IC_50_ value was determined from the dose-response curves of every cell line.

### 2.4. Phase Contrast Microscopy

A549 cells were seeded at a density of 5 × 10^4^ cells/mL into sterile culture plate and left overnight for adherence. Then, cells were incubated with helichrysetin for 24, 48, and 72 hours at 37°C and 5% CO_2_. Changes in cytomorphology of the cells which include shrinkage, detachment, and rounding were observed using phase contrast microscopy (Zeiss Axio Vert. A1).

### 2.5. Morphological Assessment by DAPI Nuclear Staining

A549 cells were incubated with helichrysetin for 24 hours. Cells were then harvested and washed with PBS. The resulting cell pellet was fixed in 4% formaldehyde. Cells were resuspended in DAPI solution (0.2 *μ*g/mL), 0.1% Triton X-100 and incubated in the dark for 5 minutes. Stained cells were spotted onto a slide and allowed to dry. Nuclear condensation and segmentation were examined under a Leica fluorescence microscope at 40x magnification, and 100 cells were counted for each sample.

### 2.6. Detection of Apoptosis by Annexin V Binding

Apoptosis detection was performed using the FITC Annexin V Apoptosis Detection Kit (BD Biosciences, USA). 8 × 10^4^ cells/mL A549 cells were plated and treated with helichrysetin for 24, 48, and 72 hours. The cells were harvested, washed with PBS, resuspended in 1× Annexin V binding buffer, and stained with annexin V and PI for 15 min at room temperature in the dark. Apoptosis was detected using Accuri C6 flow cytometer. Distribution of cell population in different quadrants was analyzed with quadrant statistics. Lower left quadrants consist of viable cells, lower right quadrants early apoptotic, and upper right quadrants late-apoptotic or necrotic cells.

### 2.7. TUNEL Assay

Apoptotic cells were detected using the APO-BrDU TUNEL Assay Kit (Invitrogen). A549 cells were treated at different time intervals: 24, 48, and 72 hours. Cells were harvested, washed, and fixed with 1% (w/v) paraformaldehyde. The cells were then centrifuged, washed, and fixed with ice-cold 70% ethanol. DNA labeling was performed according to the manufacturer's instructions, and the cells were analyzed using Accuri C6 flow cytometer.

### 2.8. Assay for Mitochondrial Membrane Potential

A549 cells (8 × 10^4^ cells/mL) were treated with helichrysetin for 24, 48, and 72 hours and stained with JC-1 (BD MitoScreen Kit) for 15 min at 37°C. Mitochondrial membrane potential was analysed using Accuri C6 flow cytometer. 

### 2.9. Cell Cycle Analysis

A549 cells were treated with helichrysetin for 24, 48, and 72 hours. The cells were harvested, washed, and fixed in 70% ethanol overnight at −20°C. Ethanol-fixed cells were pelleted, washed with ice-cold PBS, and resuspended in staining solution containing 50 *μ*g/mL PI, 0.1% Triton-X-100, 0.1% sodium citrate, and 100 *μ*g/mL RNase. After incubation for 30 min, the cells were analyzed by flow cytometer. 

### 2.10. Statistical Analysis

Results are expressed as mean ± SE from at least three independent experiments in Microsoft Excel. The Student's *t*-test was performed using SPSS Statistics 17.0 to determine statistical significance between untreated and treated groups. *P* < 0.05 was regarded as statistically significant. 

## 3. Results

### 3.1. Dose-Dependent Effect of Helichrysetin on the Growth of Cells

Table 1 shows the cytotoxic activity of helichrysetin and positive control doxorubicin on four selected cell lines. Helichrysetin showed effective cytotoxicity on all four selected cancer cell lines. This compound showed the most effective growth inhibition on cervical carcinoma cells followed by lung adenocarcinoma, breast adenocarcinoma, and colon adenocarcinoma with the IC_50_ values 31.02 ± 0.45 *μ*M (8.88 ± 0.13 *μ*g/mL), 50.72 ± 1.26 *μ*M (14.52 ± 0.36 *μ*g/mL), 97.35 ± 1.71 *μ*M (27.87 ± 0.49 *μ*g/mL), and 102.94 ± 2.20 *μ*M (29.47 ± 0.63 *μ*g/mL), respectively. Results showed significant increment of the percentage of inhibition in a dose-dependent manner (Figure 1(b)).

### 3.2. Changes in Cell and Nuclear Morphology

**Figure 2:**
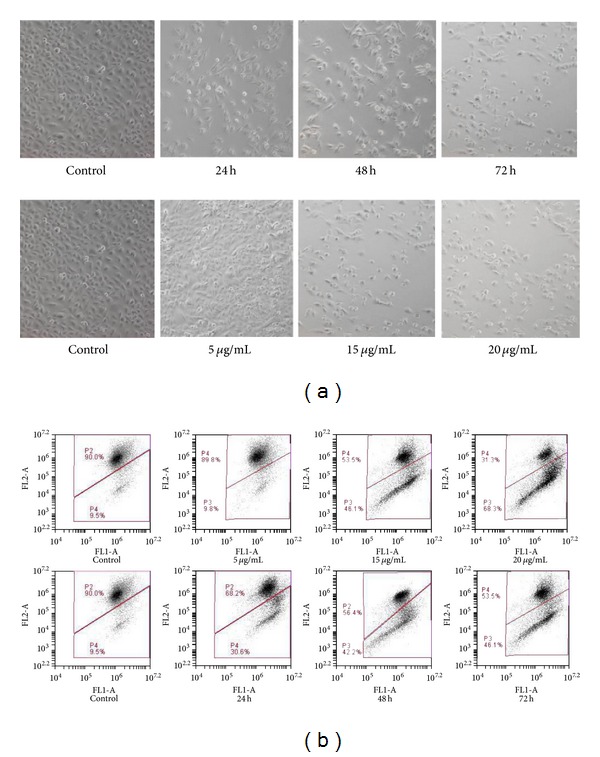
(a) Untreated control was compared with cells treated at different doses and time. Cells' morphological changes were observed under phase-contrast microscopy at 40x magnification. Cells shrinkage and rounding were observed clearly. (b) JC-1 dye was used for the analysis of cell mitochondrial membrane potential for flow cytometry as described in Section 2. Cells treated with helichrysetin were found to have lost mitochondrial membrane potential as measured by loss in fluorescence.

Phase contrast microscopy demonstrated dose- and time-dependent detachment of nonviable cells from the surface of culture plates (Figure 2(a)). Further changes in cell morphology include cell shrinkage, formation of apoptotic bodies, and membrane blebbing. Nuclear morphology changes were verified using DAPI staining in A549 cells (Figure 1(c)). Figure 1(c) shows typical nuclear morphological alterations observed through the fluorescence emission by nucleus of A549 cells. DNA samples in untreated cells were stained homogenously and less intense compared to those in treated cells. Treated cells displayed bright blue fluorescence with higher intensity than untreated cells. In addition, nuclear fragmentation and chromatin condensation which are hallmark of apoptosis were observed in helichrysetin-treated cells.

### 3.3. Helichrysetin Induces Early and Late Apoptosis in A549 Cells

**Figure 3:**
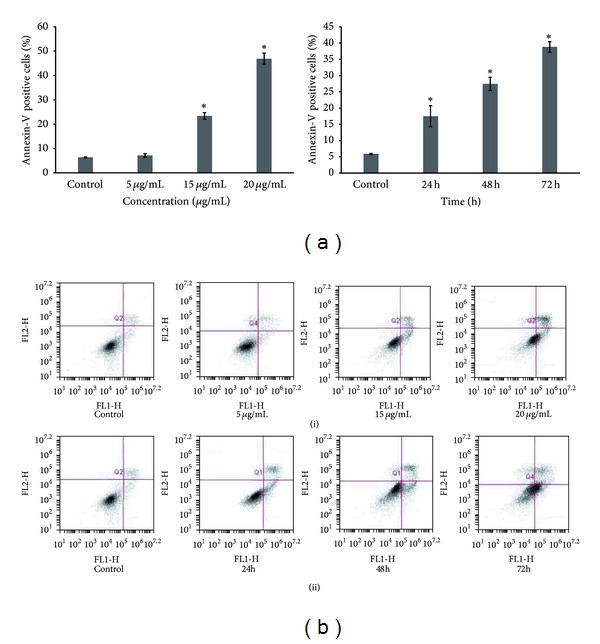
(a) Helichrysetin increases apoptotic cells significantly at 15 *μ*g/mL and 20 *μ*g/mL. Results are presented as mean ± S.E., and values of *P* < 0.05 were regarded as statistically significant compared to the untreated control. After treatment for 24 h, 48 h, and 72 h, an increase in the percentage of apoptotic cells was observed. ((b)(i)) A549 cells were treated with helichrysetin at 5 *μ*g/mL, 15 *μ*g/mL, and 20 *μ*g/mL. ((b)(ii)) A549 cells treated for 24 h, 48 h, and 72 h (bottom). Lower left quadrants show viable cells, lower right quadrants show early apoptotic cells, and upper right quadrants show late apoptotic/necrotic cells.

Figures 3(a) and 3(b) showed results of detection of apoptosis by Annexin V-PI assay. As the concentration of helichrysetin increased from 5 *μ*g/mL to 20 *μ*g/mL, the population of early apoptotic cells increased from 2.65 ± 0.31% (control) to 2.78 ± 0.21%, 14.98 ± 0.79%, and 28.55 ± 1.19%, while Annexin V/PI double positive cells increased from 3.74 ± 0.17% (control) to 4.39 ± 0.60%, 8.40 ± 1.02%, and 18.29 ± 2.58% (Figure 3(b)(i)). Results showed the increment of early and late apoptotic cells from 2.03 ± 0.18% (control) to 11.15 ± 3.53%, 15.73 ± 1.18%, and 26.92 ± 1.38% and from 3.99 ± 0.30% to 6.33 ± 0.65%, 11.70 ± 0.90%, and 11.87 ± 1.05%, respectively after treatment for 24 h, 48 h, and 72 h (Figure 3(b)(ii)). The sum of early and late apoptotic cells which make up the annexin-V positive cells showed a significant increase after treatment for 24 h, 48 h, and 72 h while the percentage of annexin-V positive cells significantly increased at 15 *μ*g/mL and 20 *μ*g/mL.

### 3.4. Helichrysetin Caused the Loss of Mitochondrial Membrane Potential

There was a loss in red fluorescence (upper quadrants) as the concentration of helichrysetin increased after treatment for 24 h, 48 h, and 72 h in a time- and dose- dependent manner (Figure 2(b)). The highest level of red fluorescence was seen in untreated control samples. The percentage of depolarized cells in the green fluorescence region (lower quadrants) significantly increased (not shown) at concentrations of 15 *μ*g/mL and 20 *μ*g/mL compared to percentage of depolarized cells in control. After treatment for 24 h, 48 h, and 72 h, the percentage of depolarized cells increased significantly. 

### 3.5. DNA Fragmentation in Helichrysetin-Treated A549 Cells

**Figure 4:**
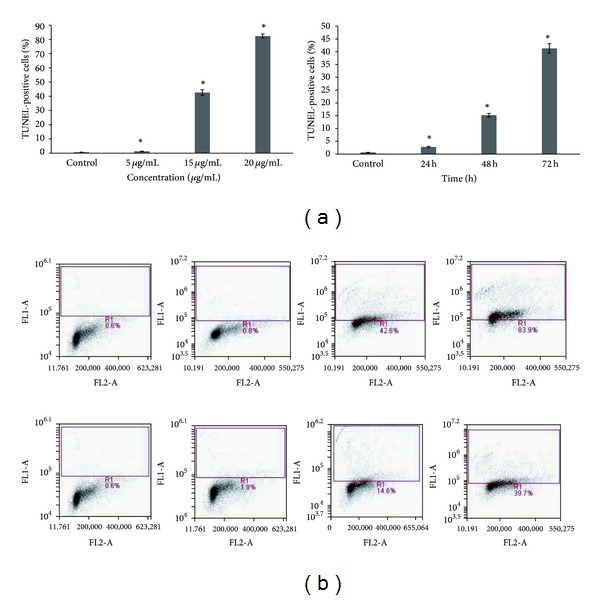
(a) TUNEL assay detects DNA fragmentation in cells. Results showed that the percentage of TUNEL-positive cells increased significantly in a dose- and time-dependent manner, presented as mean ± S.E., and values of *P* < 0.05 were regarded as statistically significant compared to the untreated control. (b) A549 cells were treated with helichrysetin at 5 *μ*g/mL, 15 *μ*g/mL, and 20 *μ*g/mL (top) and 24 h, 48 h, and 72 h (bottom). Density plots show the increase of Alexa Fluor fluorescence intensity as the dose and time increases.

A significant increase of TUNEL positive cells was observed from 0.61% to 1.28%, 42.63%, and 82.34%, when cells are treated at concentrations of 5 *μ*g/mL, 15 *μ*g/mL, and 20 *μ*g/mL (Figure 4). The percentage of TUNEL positive cells also increased significantly in a time-dependent manner from 0.61% to 2.76%, 15.16%, and 41.29%, when treated with helichrysetin at a concentration of 15 *μ*g/mL for 24 hours, 48 hours, and 72 hours.

### 3.6. Effect of Helichrysetin on Cell Cycle Distribution

**Figure 5:**
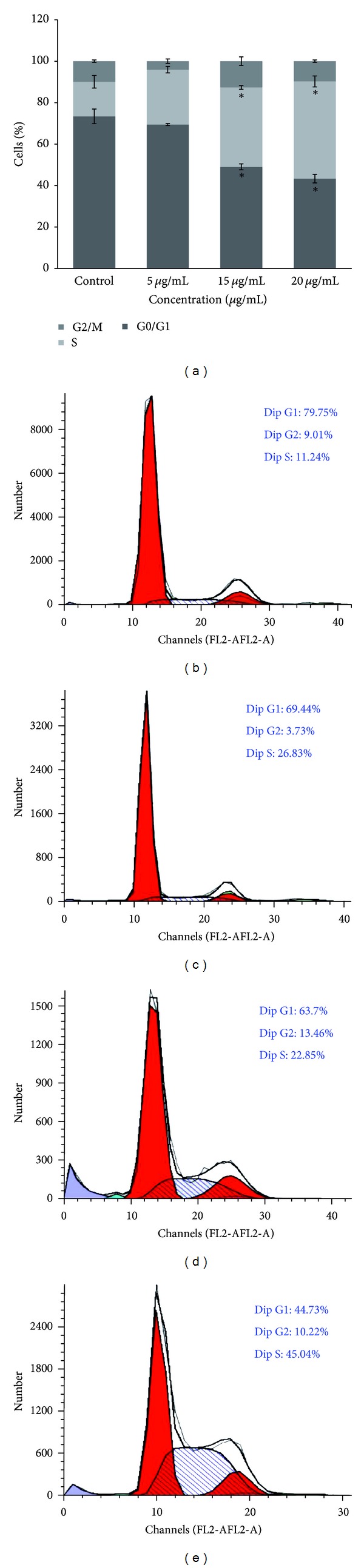
(a) Cells were treated with helichrysetin at different concentrations.After 72 hours of treatment, results showed the cell cycle distributions which have been summarized and presented as mean ± S.E., and values of *P* < 0.05 were regarded as statistically significant compared to the untreated control. *P < 0.05. (b) Cell cycle distribution of untreated cells. (c) Cell cycle distribution at 5 *μ*g/mL. (d) Cell cycle distribution at 15 *μ*g/mL. (e) Cell cycle distribution at 20 *μ*g/mL.

In A549 cells, 15 *μ*g/mL and 20 *μ*g/mL helichrysetin caused accumulation of cells in S phase, occurring simultaneously with the significant reduction of cell percentage in G0/G1 phase (Figure 5). The percentage of cells in S phase increased from 16.69 ± 2.99% (control) to 26.47 ± 1.56%, 38.29 ± 0.89%, and 46.91 ± 2.62%, when treated with increasing concentrations of helichrysetin (5–20 *μ*g/mL).

## 4. Discussion

In this study, we report the mechanisms of apoptosis induced by a natural compound, helichrysetin on human lung adenocarcinoma, A549 and the cytotoxic activity on four selected cancer cell lines. Previous studies have shown effective cytotoxic activity of helichrysetin on several cancer cell line [5, 6, 9]. To the best of our knowledge, this study represents a first report of the cytotoxic activity on human lung adenocarcinoma and human cervical carcinoma cancer cell lines. Here, we have shown the biochemical and molecular mechanisms of apoptosis induced by helichrysetin in cancer cells. 

Based on our data (Table 1), helichrysetin showed a good cytotoxic effect on all the four selected cancer cell lines with the highest cytotoxic activity on human cervical carcinoma, Ca Ski with IC_50_ of 31.02 ± 0.45 *μ*M (8.88 ± 0.13 *μ*g/mL) followed by the human lung adenocarcinoma, and A549 with IC_50_ of 50.72 ± 1.26 *μ*M (14.52 ± 0.36 *μ*g/mL). A direct-acting natural compound is considered active against cancer cells *in vitro* when the IC_50_ is within the concentration range of 1–50 *μ*M [19]. Dose- (Figure 1(b)) and time-dependent (not shown) cytotoxicity of helichrysetin on all four cancer cell lines was observed. This suggested that treatment with helichrysetin inhibited the growth and reduced the viability of these cells. The induction of apoptosis has been described as a standard and best strategy in anticancer therapy [20, 21]. 

Phase-contrast microscopy revealed the early stages of apoptosis which are characterized by the shrinkage of cells, blistering, and membrane blebbing [22, 23]. As seen in the time-dependent treatment (Figure 2(a)), cells started to detach from the surface of the culture plates. Apoptosis is also characterized by the condensation of nuclear chromatin followed by the eventual breakup of the chromatin leading to nuclear fragmentation [23]. After treatment with 15 *μ*g/mL helichrysetin for 24 hours, A549 cells showed signs of nuclear fragmentation and chromatin condensation (Figure 1(c)). 

 Detection of early and late apoptosis was performed with Annexin-V staining. Annexin-V binds to the externalized phosphatidylserine (PS) of apoptotic cells [24]. Changes on the surface of apoptotic cells include the externalization of PS which is a phospholipid present in the inner leaflet of plasma membrane [25, 26]. When apoptosis occurs, the asymmetry of phospholipid is broken, and PS is translocated to the outer leaflet of the plasma membrane [26]. Cells in late stage of apoptosis or necrosis are both annexin and PI positive [27]. The occurrence of early and late apoptosis is validated by the increase of Annexin-V positive cells in dose- and time-dependent experiments (Figure 3). 

Impairment of mitochondria membrane is involved in the induction of apoptosis [28]. Many researchers have demonstrated the changes in mitochondrial structure and the collapse of mitochondrial membrane potential [29], ΔΨm prior to apoptosis. A membrane-permeable cationic fluorochrome, JC-1, was used to evaluate the mitochondrial membrane polarization in A549 cells [30]. When the mitochondrial membrane potential collapse, there will be a loss of JC-1 aggregates, and the dye will move out of the mitochondria resulting in the drop of red fluorescence [31]. Treatment of A549 cells with different concentrations of helichrysetin and at different time points resulted in a drop of red fluorescence in a dose- and time-dependent manner (Figure 2(b)). The ability of helichrysetin to induce apoptosis is also supported by measuring the DNA damage in cells. As shown in Figure 4, TUNEL-positive cells were detected by flow cytometry which indicates apoptotic cells with fragments of DNA. During apoptosis, DNA strand breaks will expose the 3′OH ends which act as sites for the addition of 5-bromo-2′-deoxyuridine 5′-triphosphate (BrdUTP) [32]. The incorporation was detected using Alexa Fluor 488 dye-labeled anti-BrdU antibody [33]. 

In this study, we have found that helichrysetin induced apoptosis in A549 based on the evidence given such as a significant increase in the externalization of phosphatidylserine, mitochondrial membrane depolarization, and DNA fragmentation which are the features of apoptotic cells [34] mostly at the concentrations of 15 *μ*g/mL and 20 *μ*g/mL. This can be further supported by results of cell cycle distribution which showed the accumulation of cells in the S phase and the decrease of cell percentage in the G0/G1 phase. Accumulation of cells in S phase may have contributed to the high level of apoptosis in A549 cells [35]. S phase blockade is now a checkpoint that inhibits the replication on damaged DNA which caused a decrease in cell survival [36, 37]. Hence, the data suggested that helichrysetin altered the cell cycle in a dose-dependent manner, and this could explain the observed correlation between cell growth inhibition, cell death, and cell cycle blockade [38].

## 5. Conclusion

 Our study clearly demonstrates that helichrysetin possess strong inhibitory effects on cell growth and is capable of inducing apoptosis in A549 cells. Helichrysetin also appears to affect the cell cycle in a manner that favors apoptosis. The present findings provide valuable information in the development of natural compounds for use in cancer therapy.

## Figures and Tables

**Table 1:** IC_50_ values of helichrysetin and doxorubicin on selected cancer cells.

Cells	Type	Helichrysetin	Doxorubicin
		IC_50_ (*μ*M)	IC_50_ (*μ*M)
A549	Lung adenocarcinoma	50.72 ± 1.26 (14.52 ± 0.36 *μ*g/mL)	1.10 ± 0.02 (0.19 ± 0.01 *μ*g/mL)
Ca Ski	Cervical carcinoma	31.02 ± 0.45 (8.88 ± 0.13 *μ*g/mL)	0.35 ± 0.02 (0.07 ± 0.01 *μ*g/mL)
HT-29	Colon adenocarcinoma	102.94 ± 2.20 (27.87 ± 0.49 *μ*g/mL)	0.48 ± 0.08 (0.60 ± 0.01 *μ*g/mL)
MCF-7	Breast adenocarcinoma	97.35 ± 1.71 (29.47 ± 0.63 *μ*g/mL)	0.13 ± 0.02 (0.26 ± 0.04 *μ*g/mL)

IC_50_: concentration that causes 50% of cell growth inhibition. Results are shown as mean ± S.E. from three independent experiments.
